# Classical lepidopteran wing scale colouration in the giant butterfly-moth *Paysandisia archon*

**DOI:** 10.7717/peerj.4590

**Published:** 2018-04-11

**Authors:** Doekele G. Stavenga, Hein L. Leertouwer, Andrej Meglič, Kazimir Drašlar, Martin F. Wehling, Primož Pirih, Gregor Belušič

**Affiliations:** 1 Department of Computational Physics, University of Groningen, Groningen, Netherlands; 2 Department of Biology, University of Ljubljana, Ljubljana, Slovenia; 3 Air Force Research Laboratory, Eglin Air Force Base, Eglin, USA

**Keywords:** Pigments, Spectrophotometry, Ommochrome, Thin films, Melanin

## Abstract

The palm borer moth *Paysandisia archon* (Castniidae; giant butterfly-moths) has brown dorsal forewings and strikingly orange-coloured dorsal hindwings with white spots surrounded by black margins. Here, we have studied the structure and pigments of the wing scales in the various coloured wing areas, applying light and electron microscopy and (micro)spectrophotometry, and we analysed the spatial reflection properties with imaging scatterometry. The scales in the white spots are unpigmented, those in the black and brown wing areas contain various amounts of melanin, and the orange wing scales contain a blue-absorbing ommochrome pigment. In all scale types, the upper lamina acts as a diffuser and the lower lamina as a thin film interference reflector, with thickness of about 200 nm. Scale stacking plays an important role in creating the strong visual signals: the colour of the white eyespots is created by stacks of unpigmented blue scales, while the orange wing colour is strongly intensified by stacking the orange scales.

## Introduction

Of the lepidopteran insects (moths and butterflies), especially the diurnally active butterflies are universally treasured for their often bright and colourful patterning. This is also the case for some diurnal moth species, but moths generally feature inconspicuous colourations, obviously related to their nocturnal lifestyle. The colouration pattern is due to the lattice of scales that cover the wings like shingles on a roof (Nijhout, 1991; Ingram & Parker, 2008; Kinoshita, 2008). The colour of the individual wing scales is usually determined by pigments, but often the structural organization of the scales plays a prominent role (Vukusic & Sambles, 2003; Yoshioka & Kinoshita, 2007). Subtle colouration effects are achieved by combining pigmentary and structural colouration methods (Yoshioka & Kinoshita, 2006; Trzeciak et al., 2012; Wilts et al., 2015).

The structure of lepidopteran wing scales has been investigated in extensive detail by Ghiradella (Ghiradella, 1991; Ghiradella, 1998; Ghiradella, 2010). The chitinous wing scales are organised into two laminae, i.e., a more or less flat, thin lower lamina and a highly structured upper lamina, consisting of rows of parallel ridges with cross-ribs in between. The two laminae are connected by trabeculae, pillar-like elements serving as mechanical struts and spacers.

As the lower lamina thickness is of the order of 200 nm, it acts as an optical thin film, causing a distinct reflectance in a restricted wavelength range, critically depending on the precise thickness. Quite differently, the upper lamina is commonly highly convoluted and thus acts as a broad-band diffuser. If the scale contains a dense pigmentation, the light reflected and scattered by the scale structures is selectively filtered, so that then a distinct pigmentary colouration remains. However, when the scale is unpigmented, the lower lamina determines the scale colour (Stavenga, Leertouwer & Wilts, 2014a; Stavenga, Leertouwer & Wilts, 2014b).

Simple as well as very sophisticated modulations on this theme have been discovered in various butterfly species. For instance, in a certain scale type of the papilionid butterfly *Graphium sarpedon* the upper lamina is fully flattened and pressed against the lower lamina so that the two laminae together form a (thickened) thin film (Stavenga et al., 2012). On the other hand, extremely complex multilayer and gyroid photonic systems have been found to exist in lycaenid butterflies (Michielsen & Stavenga, 2008; Saranathan et al., 2010; Wilts et al., 2017a), and furthermore, the spectral reflectance of such 3D photonic crystals tuned by pigmentary filtering has been recently unraveled in detail (Wilts et al., 2012; Wilts, IJbema & Stavenga, 2014).

The pigments colouring the lepidopteran wing scales characteristically differ between butterfly families, except for melanin pigment that is universally shared (Nijhout, 1991). Pterins are the pigments generally encountered in Pieridae (Descimon, 1975; Wijnen, Leertouwer & Stavenga, 2007). A quite widespread pigment is kynurenine, causing a yellow colour. It is a precursor of the ommochromes, generally identified in Nymphalidae (Nijhout, 1997; Reed, McMillan & Nagy, 2008; Wilts et al., 2017b). Kynurenine bound to N-β-alanyl-dopamine (NBAD) creates the papiliochromes found in Papilionidae (Umebachi, 1985; Nijhout, 2010; Nishikawa et al., 2013; Wilts, IJbema & Stavenga, 2014). Some papilionids use blue–green bile pigments, neopterobilins (Choussy et al., 1973), which were also claimed to exist in Geometrinae and other moths (Barbier, 1986). However, the Geometrinae were reported to have a novel green pigment type, geoverdin (Cook et al., 1994).

**Figure 1:**
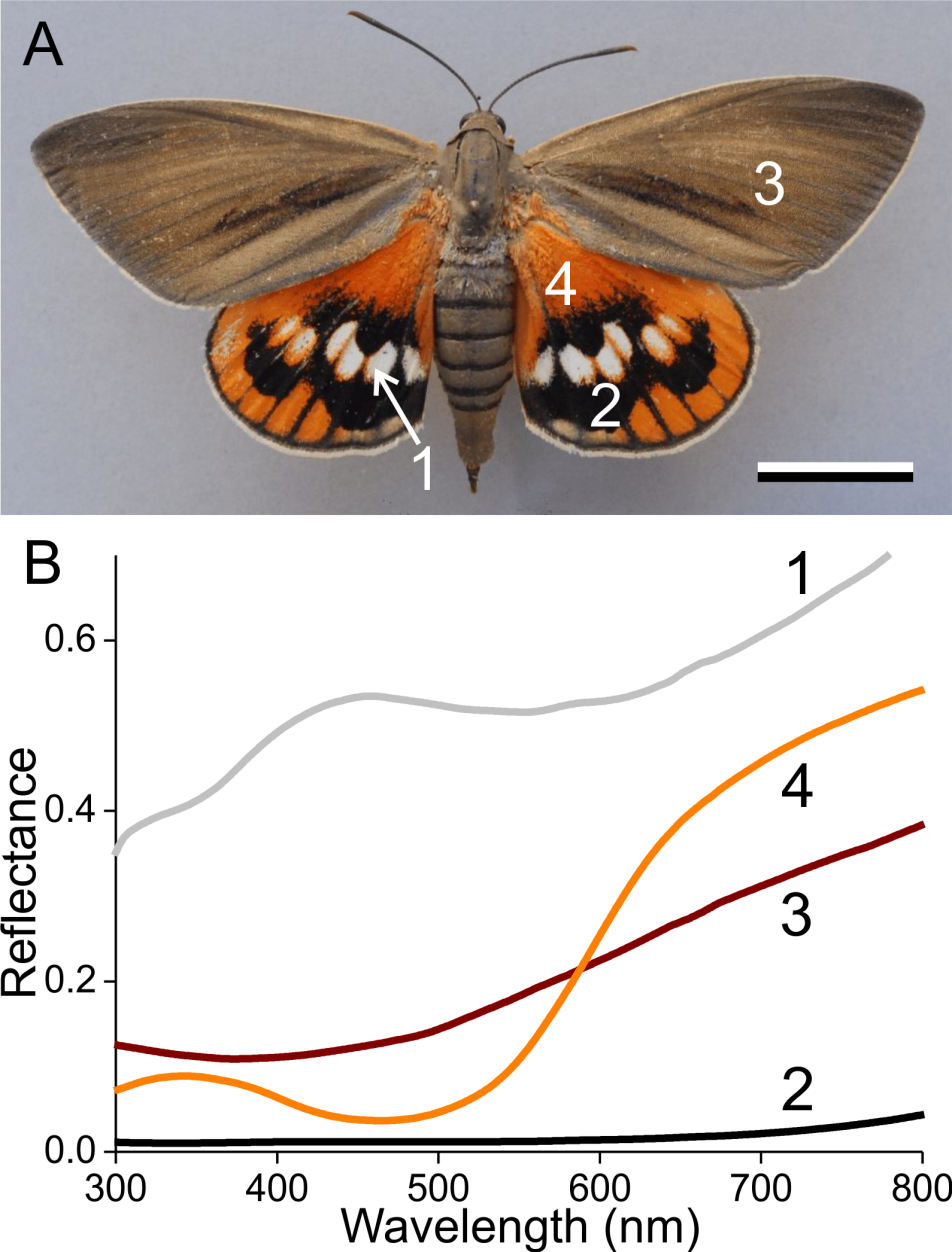
The palm borer moth *Paysandisia archon* and wing colouration. (A) A female *P. archon* showing drab brown forewings and colourful hindwings (dorsal view, photo courtesy of Dr. Jean-François Germain). The numbers indicate the measurement location of the spectra; scale bar: 2 cm. (B) Reflectance spectra of the white (1), black (2), brown (3) and orange (4) wing areas.

Virtually all studies on the colouration of Lepidoptera have been performed on butterflies. Those devoted to moths concern the extremely colourful dayflying moths, i.e., the swallowtail moths *Urania fulgens* and *U. leilus* and the sunset moth *Chrysiridia ripheus* (Uraniinae). These structurally coloured moths apply advanced multilayer optics in sometimes strongly curved scales, resulting in distinct colour mixing and polarization effects (Onslow, 1923; Süffert, 1924; Yoshioka & Kinoshita, 2007; Yoshioka et al., 2008). Investigations into pigmentary coloured moths are scarce, however. Melanins doubtless generally occur in moths, but the pigments common in butterflies have not been specifically identified. Here we investigate the colouration of the palm borer moth *Paysandisia archon* (Fig. 1A), like the diurnally active uraniines a day-flying moth, which appears to have a rather straightforward colour patterning with wing scales that apply a universal, classical lepidopteran colouration method.

## Materials and Methods

### Spectrophotometry

Reflectance spectra of different areas of the wings of female palm borer moths, obtained from CIRAD/CSIRO (Montpellier), were measured with a bifurcated probe (Avantes FCR 7-UV-200), using an AvaSpec 2048-CCD detector array spectrometer (Avantes, Apeldoorn, The Netherlands). The light source was a deuterium-halogen lamp (AvaLight D(H) S), and the reference was a white diffuse reflectance tile (Avantes WS-2). Reflectance spectra of isolated scales were measured with a microspectrophotometer (MSP). The scales were attached to a glass micropipette using Bison glass kit (hardening under UV light). Care was taken that the glue remained restricted to a very local area and did not affect the colour of other scale areas. The MSP was a Leitz Ortholux microscope (Leitz, Wetzlar, Germany) with objective an Olympus 20x, NA 0.46 (Olympus, Tokyo, Japan) and as light source a xenon arc lamp. The area measured with the MSP was a square with edge length 5–10 µm, determined by a square diaphragm in the microscope’s image plane, which was in turn imaged at the entrance of an optical fibre connected to the detector array spectrometer; the white diffuse reflectance tile was also here used as a reference. Due to the glass optics in the microscope, the MSP spectra were limited to wavelengths >350 nm. The applied epi-illumination had an about normal direction, and as the scale’s thin film reflections are directional and the reference is a diffuser, this caused overestimated reflectance values. Absorbance spectra of isolated scales, 5–10 scales of each type, immersed in immersion oil (*n* = 1.515), were also measured with the MSP. Absorbance spectra of scale pigment extracts were measured in quartz cuvettes using a custom-built setup. The pigments were extracted by putting wing fragments into a solution of 50:1 methanol: 1 M hydrochloric acid (Bolognese et al., 1988; Wilts et al., 2017b).

### Electron microscopy

Parts of wings with differently coloured scales were fixed for 45 min in 2.5% glutaraldehyde and 2% formaldehyde in 0.1 M Na-cacodylate buffer (pH 7.2). The specimens were rinsed in 0.1 M Na-cacodylate and distilled water, dehydrated in graded ethanol (50–100% in 10% steps) series, infiltrated with propylene oxide and embedded in Spurr’s resin (SPI Supplies, West Chester, PA, USA). Ultrathin sections were made with a diamond knife (Histo, Diatom, Switzerland), double-stained with uranyl acetate and lead citrate and observed with a transmission electron microscope CM 100 (Philips, Eindhoven, The Netherlands) and imaged with an Orius 200 (Gatan, Pleasanton, CA, USA) camera. For scanning electron microscopy (SEM) wings were air-dried. Individual scales were attached to adhesive conductive carbon discs (SPI, USA) on metal holders. Specimens were coated with platinum (layer thickness 3 nm) and observed with a field-emission scanning electron microscope JSM-7500F (JEOL, Tokyo, Japan).

### Imaging scatterometry of single wing scales

For investigating the spatial reflection characteristics of the scales, we performed imaging scatterometry (Stavenga et al., 2009; Wilts, Leertouwer & Stavenga, 2009). A scale attached to a glass micropipette was positioned at the first focal point of the ellipsoidal mirror of the imaging scatterometer. The scatterograms were obtained by focusing a white light beam with a narrow aperture (<5°) onto at a small circular area (diameter 13 µm), and the spatial distribution of the far-field scattered light was then monitored. The exposure times of the scatterograms were appropriately adjusted so as to obtain a contrastful image.

### Modelling the reflectance spectra of thin films and scale stacks

Reflectance spectra of chitinous optical thin films (*R*_*f*_) in air were calculated for normally incident light using an expression derived from the classical Airy formula (Stavenga, 2014):

(1) \begin{eqnarray*}{R}_{f}=2{R}_{b}(1-\cos \nolimits \psi )/(1+{R}_{b}^{2}-2{R}_{b}\cos \nolimits \psi )\end{eqnarray*}

where *ψ* = 4*πn*_*c*_*d*∕*λ*; *d* is the thickness of the thin film layer, *λ* is the wavelength, *n*_*c*_ = *A*_*c*_ + *B*_*c*_*λ*^−2^ is the refractive index of the chitin medium, with *A*_*c*_ = 1.517 and *B*_*c*_ = 8,800 nm^2^ (Leertouwer, Wilts & Stavenga, 2011), and *R*_*b*_ = [(*n*_*c*_ − 1)∕(*n*_*c*_ + 1)]^2^ is the reflectance of the air–chitinous thin-film boundary for normally incident light.

The reflectance spectra of a pile of scales stacked on the wing were calculated with a transfer matrix formalism, where for a stack of *N* layers the transfer matrix for each layer is given by

(2) \begin{eqnarray*}{M}_{i}={T}_{i}^{-1} \left[ \begin{array}{@{}cc@{}} \displaystyle {T}_{i}^{2}-{R}_{i}^{2}&\displaystyle {R}_{i}\\ \displaystyle -{R}_{i}&\displaystyle 1 \end{array} \right] \end{eqnarray*}

with *R*_*i*_ and *T*_*i*_ the reflectance and transmittance of layer *i* = 1, …*N*. The reflectance of the total stack follows from the product matrix

(3) \begin{eqnarray*}{M}_{s}={M}_{N}{M}_{N-1}\ldots {M}_{1}={T}^{-1} \left[ \begin{array}{@{}cc@{}} \displaystyle {T}^{2}-{R}_{1N}{R}_{N1}&\displaystyle {R}_{N1}\\ \displaystyle -{R}_{1N}&\displaystyle 1 \end{array} \right] \end{eqnarray*}

where *R*_1*N*_ is the reflectance when light is incident at layer 1, and *R*_*N*1_ is the reflectance for light incident at layer *N*; *T* is the transmittance of the stack, which is the same for both ways of illumination (for details, see Stavenga & Van der Kooi, 2016).

This transfer matrix procedure holds for a stack of plates that is illuminated with incoherent light, which is applicable here, because neither the distance between the upper and lower lamina nor the distance between stacked scales is sufficiently constant to cause interference effects. The procedure thus fundamentally differs from the transfer matrix method for calculating the reflectance of an optical multilayer, i.e., a stack of layers illuminated with coherent light (see e.g., Stavenga, 2014).

## Results

### Wing colours and reflectance spectra

Palm borer moths have dorsal forewings with an overall brown colour. The mainly orange dorsal hindwings feature prominent white spots surrounded by black areas (Fig. 1A). We measured the reflectance spectra of the various areas with a bifurcated probe (Fig. 1B). The white spots (#1) have a broad-band reflectance spectrum with a slight hump in the blue (between 400–500 nm). The reflectance of the black areas (#2) is obviously very low throughout the whole visible wavelength range, including the UV, and the reflectance spectra of the brown (#3) and orange (#4) areas characteristically increase with increasing wavelengths.

**Figure 2:**
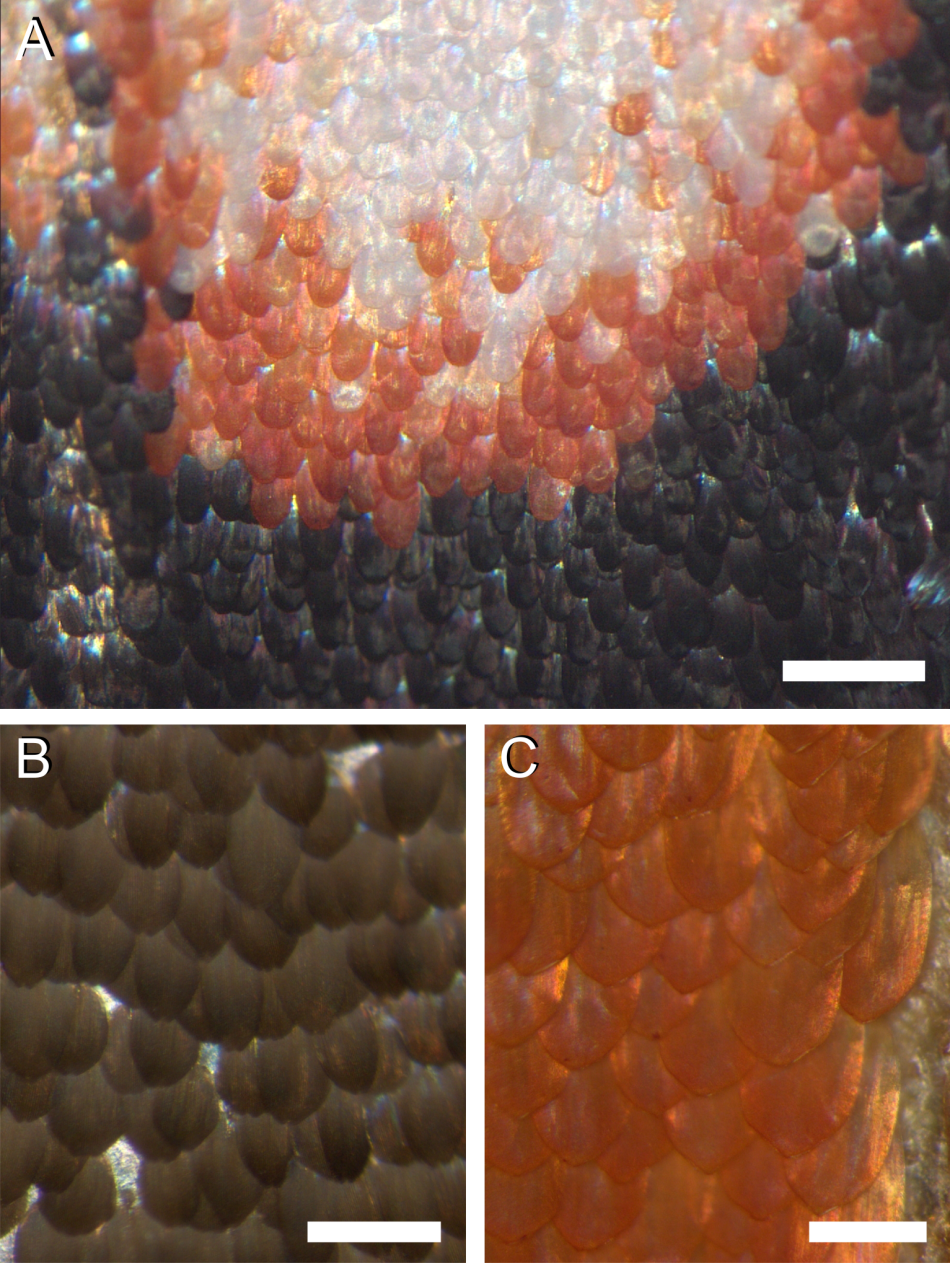
Photographs of the irregular distribution of the scales on the wing of *P. archon*. (A) Transition area of a white spot via an orange rim to the black surroundings (near the arrowhead of #1 of Fig. 1A). (B) An area of the forewing (near #3 of Fig. 1). (C) An orange hindwing area (at #4 of Fig. 1). Scale bars: (A) 0.5 mm; (B, C) 0.25 mm.

The colours are created by the wing scales (Fig. 2). Whereas generally in butterflies the scales are arranged in neat rows of cover scales overlapping ground scales (e.g., Ghiradella, 1998; Nijhout, 2010), in the palm borer moth this regular arrangement cannot be discerned. The scales of a certain type overlap each other rather randomly, in the border areas of the white spots (Fig. 2A) as well as in the homogeneously coloured brown and orange areas (Figs. 2B, 2C).

### Scale pigmentation and absorbance spectra

**Figure 3:**
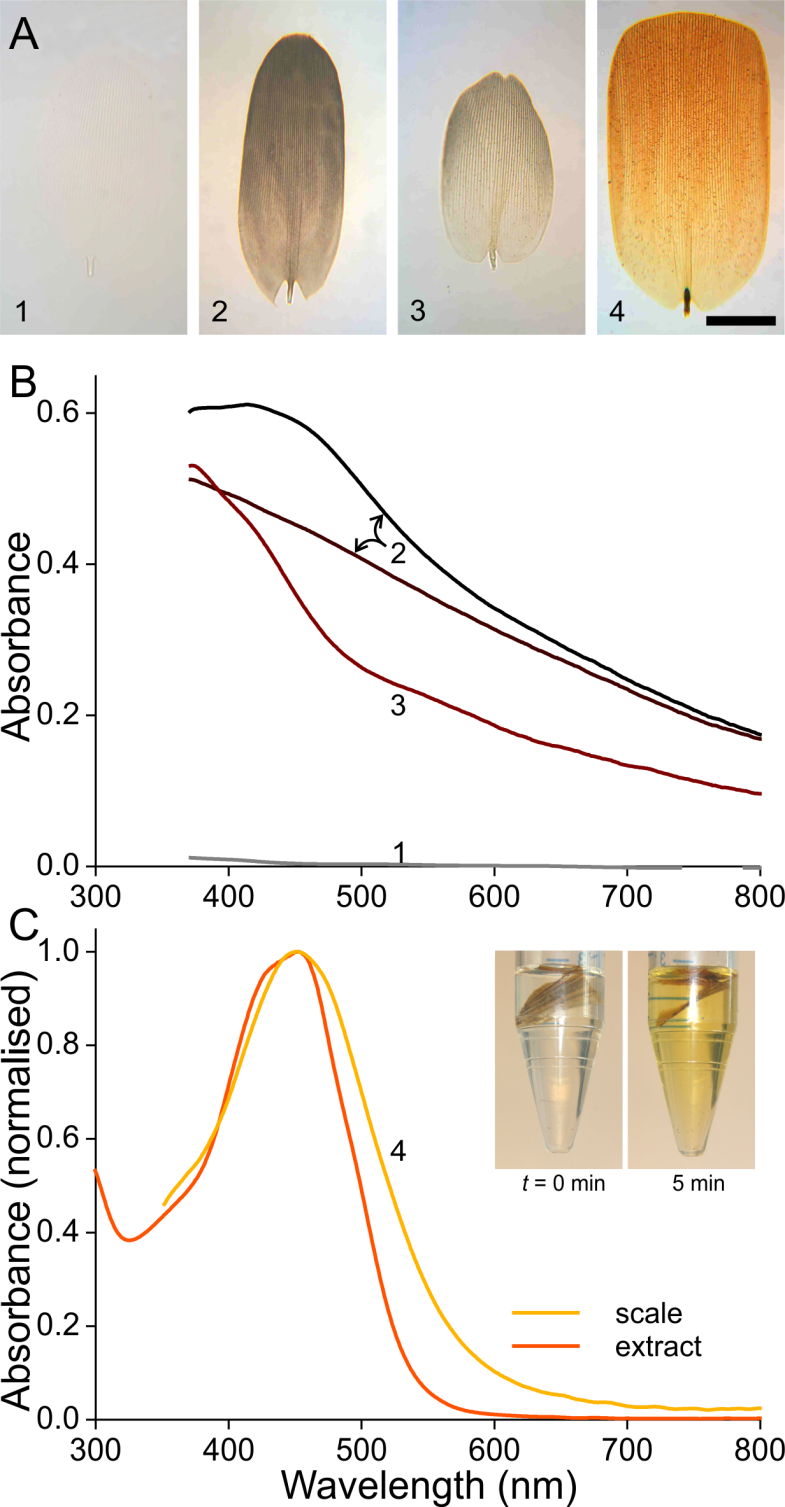
Absorbance of wing scale pigments. (A) Transmission photographs of wing scales isolated from the areas 1–4 of Fig. 1, immersed in oil; scale bar: 50 µm. (B) Absorbance spectra of two black scales (area #2 of Fig. 1), of a brown forewing scale (area #3), and of a scale from the white area (#1). (C) Normalised absorbance spectra of an orange scale immersed in oil (#4) and of an extract of wing pieces; inset: wing pieces immediately after being put into the acidified methanol solution and 5 min later.

To investigate the origin of the scale colours, we detached the variously coloured scales from the wings by pressing wing pieces onto a microscope slide, applied then a drop of immersion oil on the loosened scales, and subsequently sealed them with a cover slip. Figure 3A shows transmission light micrographs of scales from the white, black, brown and orange areas, respectively. Figures 3B and 3C present absorbance spectra measured from small areas of the immersed isolated scales.

The white scale is virtually perfectly transparent (Fig. 3A, #1) and thus has negligible absorbance (Fig. 3B, #1). The absorbance of the black and brown scales decreases with increasing wavelength, indicating the ample presence of melanin (Figs. 3A, 3B, #2, 3; Stavenga, Leertouwer & Wilts, 2014a; Stavenga, Leertouwer & Wilts, 2014b). The spectral shape varies between similar-coloured scales, however, as shown by the spectra from two black scales (Fig. 3B, #2). The spectra deviate in the shorter wavelength range, which indicates the presence of another pigment, in addition to the prominent melanin. The brown forewing scale (Figs. 3A, 3B, #3) has a lower concentration of melanin, but its absorbance spectrum also here suggests the presence of an additional short-wavelength absorbing pigment. The orange scale (Figs. 3A, 3C, #4) strongly absorbs in the short-wavelength range, with absorbance peak wavelength ∼450 nm. The measured absorbance peak values varied considerably, between 0.4 and 1.0, across the scale area and between different scales (the average 0.6 was used in modeling; see below)

The shape of the orange scale’s absorbance spectrum resembles that of the absorbance spectra of known ommochromes (Bolognese et al., 1988; Nijhout, 1997). These are known to be extractable by solutions of acidified methanol, and indeed, orange wing pieces put into such solutions rapidly turned yellow (Fig. 3C, inset; see ‘Materials and Methods’). The measured absorbance spectra of the yellowed solutions corresponded well with the absorbance spectra of the immersed scales. The slightly broader spectrum of the immersed scale is presumably due to scale inhomogeneities (Fig. 3C).

We noted above that the absorbance spectra of the black and brown scales had different shapes, especially in the short-wavelength range (Fig. 3B). Comparing the spectral deviations with the blue-violet peaking absorbance spectra of Fig. 3C suggests that the latter scales contain various amounts of ommochrome pigment next to melanin.

### Scale anatomy and thin film optics

**Figure 4:**
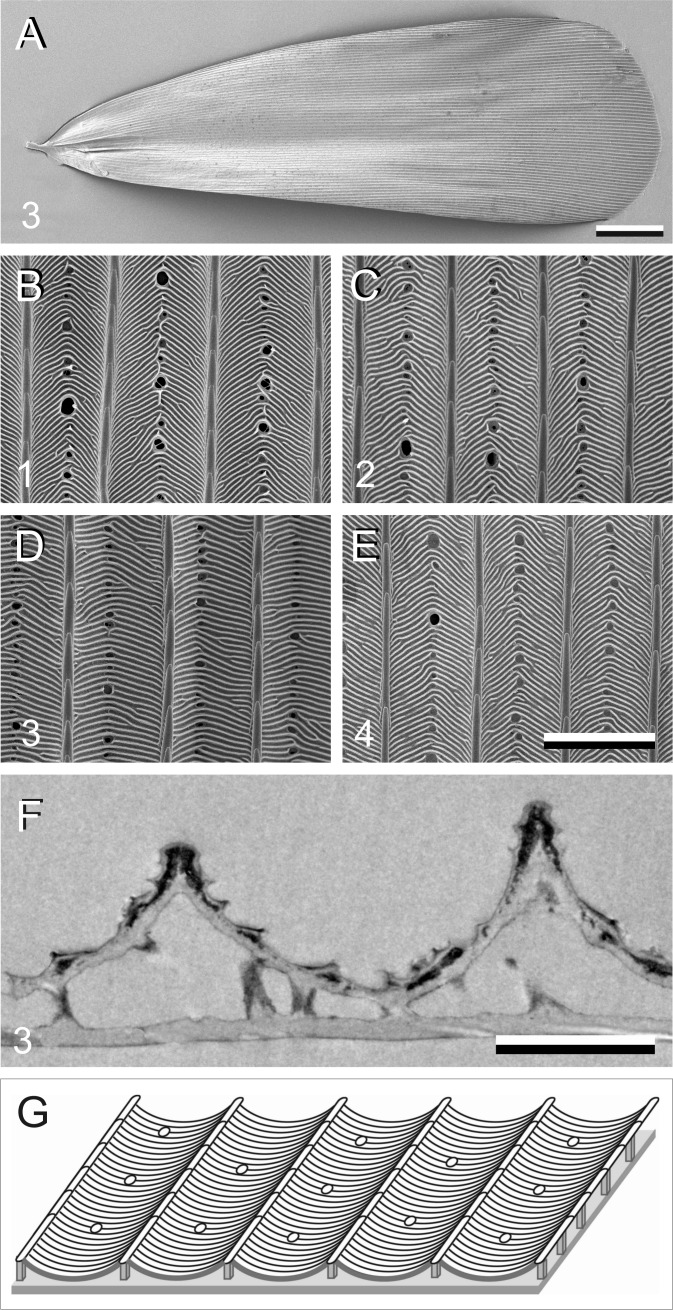
Electron micrographs of *P. archon* wing scales. (A) Scanning electron micrographs of the upper (abwing) surface of a brown scale (3); scale bar: 100 µm. (B–E) Scanning electron micrographs of the abwing surface of a white (1), black (2), brown (3), and orange scale (4), showing a few ridges and the connecting crossribs with minimal windows; scale bar: 5 µm. (F) Transmission electron micrograph of a brown scale (3); scale bar: 2 µm. (G) Diagram of a scale showing the flat and thin lower lamina supporting via pillar-like trabeculae the highly structured upper lamina with the array of parallel ridges.

In addition to pigments, structural effects can also contribute to the scale colouration. We therefore investigated the scale anatomy by performing scanning and transmission electron microscopy (Fig. 4). The scales appeared to have the classical lepidopteran organization of a flat lower lamina and a highly structured upper lamina, which consists of an array of parallel ridges, spaced apart by ∼4 µm, with slightly overlapping lamellae. The crossribs, which connect the ridges, are extremely numerous in all four scale types investigated, leaving minimally-sized or no windows (Figs. 4B–4E). The transmission electron micrograph shows a brown scale, with black stain in the upper lamina, indicating the presence of melanin there (Fig. 4F). Figure 4G shows schematically the structure of a scale.

**Figure 5:**
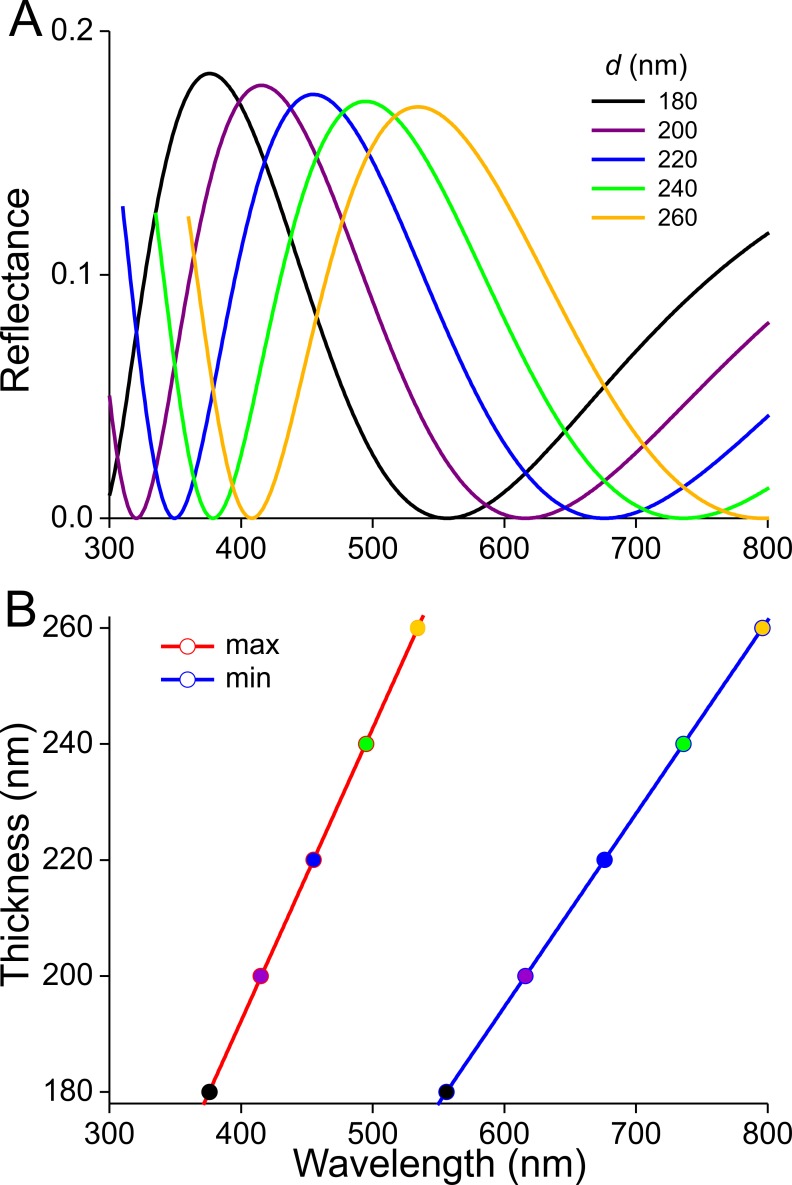
Reflectance characteristics of thin films. (A) Reflectance spectra of normally illuminated, chitinous thin films with thickness 180–260 nm. (B) Maximum and minimum reflectance wavelengths as a function of thin film thickness.

Previous studies demonstrated that the lower lamina is a more or less flat and thin plate acting as an optical thin film. The thickness of the scale of Fig. 4F is ∼240 nm. We therefore calculated reflectance spectra for thin films made of butterfly chitin having a thickness 180–260 nm (Fig. 5A). The spectra have peak wavelengths in the range 375–535 nm and valleys at 550–800 nm. Figure 5B shows the functional relationship of the maximum and minimum reflectance wavelengths with the thin-film thickness. These theoretical spectra will be used in the interpretation of the reflectance spectra measurement presented below.

### Scattering and spectral characteristics of the scales

**Figure 6:**
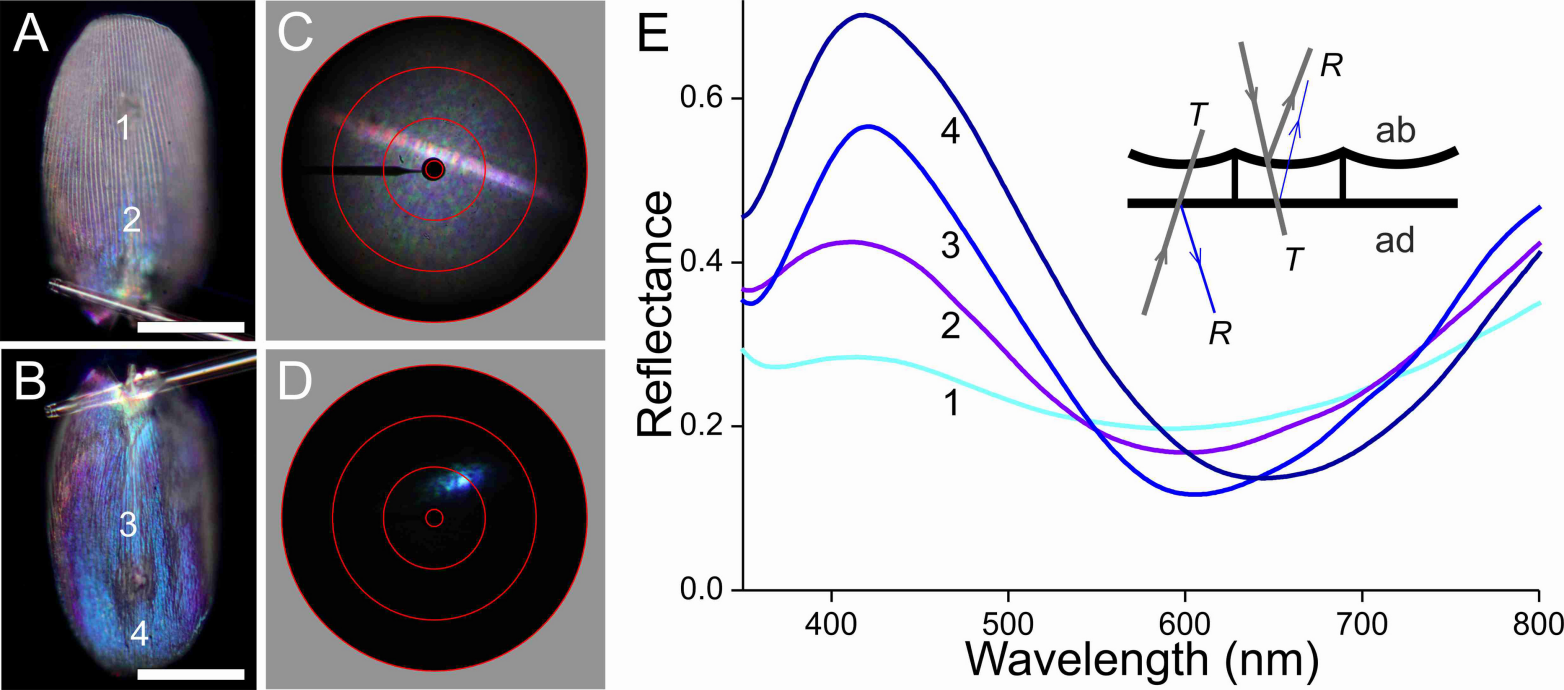
Scale of a white hindwing area (Fig. 1, #1) glued to a glass micropipette. (A, B) Photographs of the abwing (upper) and adwing (lower) sides (scale bars: 50 µm). (C, D) Scatterograms of the two sides (red circles indicate scattering angles of 5°, 30°, 60°, 90°). (E) Reflectance spectra measured from the numbered locations in (A and B). Inset: diagram of the reflection (*R*) and transmission (*T*) processes of the white wing scale. With white light incident at the abwing side (ab), part of the light is scattered by the upper lamina and part of the transmitted light is reflected by the lower lamina. White light incident at the adwing side results in a mainly blue reflection.

**Figure 7:**
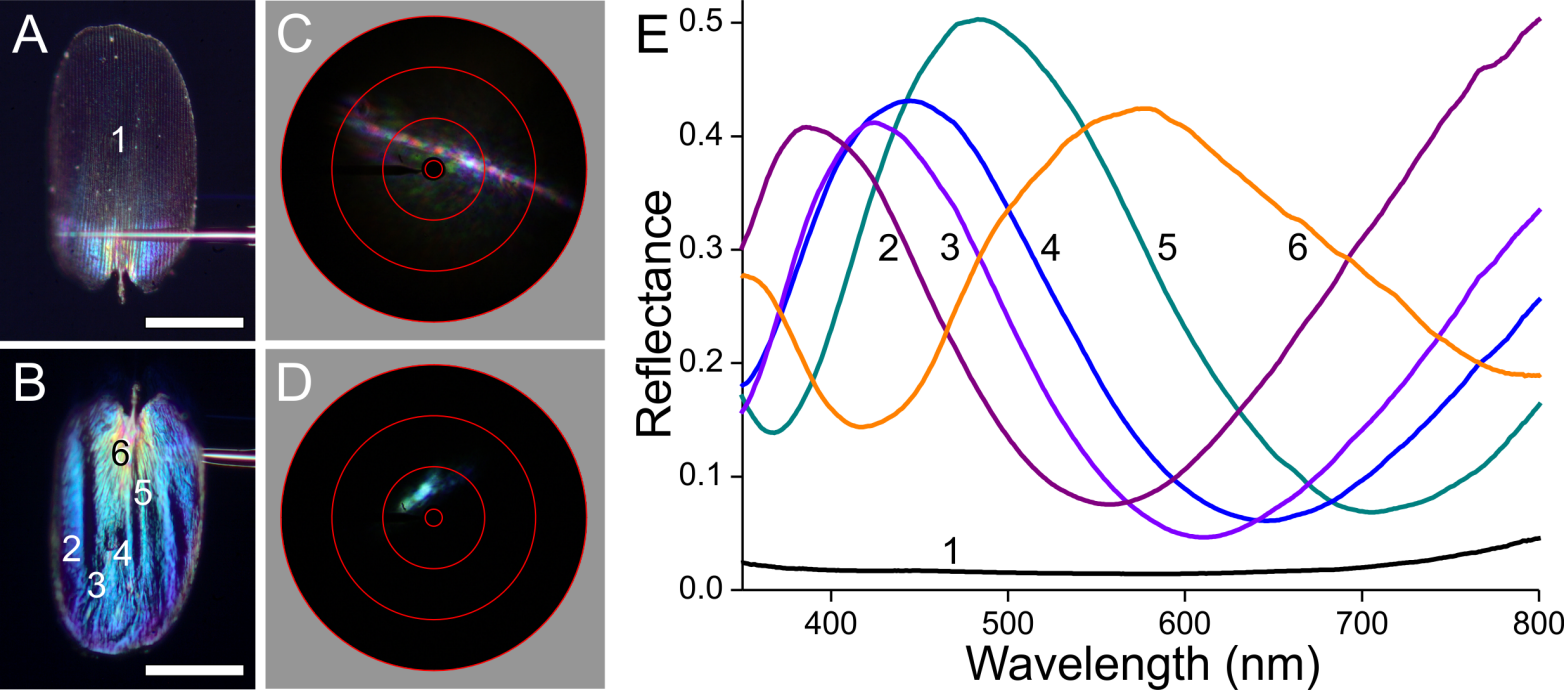
Scale of a black hindwing area (Fig. 1, #2). (A, B) Photographs of the abwing and adwing sides (scale bars: 50 µm). (C, D) Scatterograms of the two sides. (E) Reflectance spectra of the numbered locations in (A) and (B).

**Figure 8:**
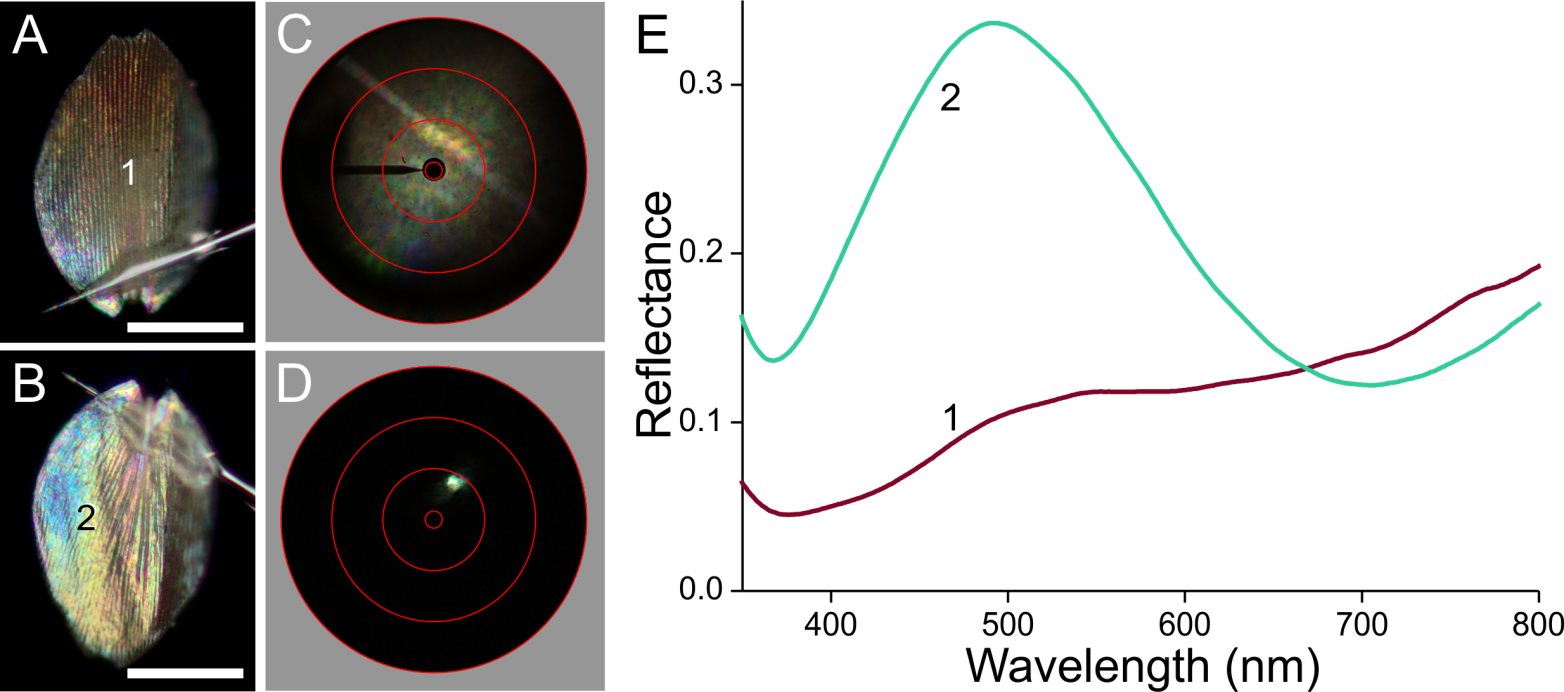
Scale of a brown forewing area (Fig. 1, #3). (A, B) Photographs of the abwing and adwing sides (scale bars: 50 µm). (C, D) Scatterograms of the two sides. (E) Reflectance spectra of the numbered locations in (A) and (B).

**Figure 9:**
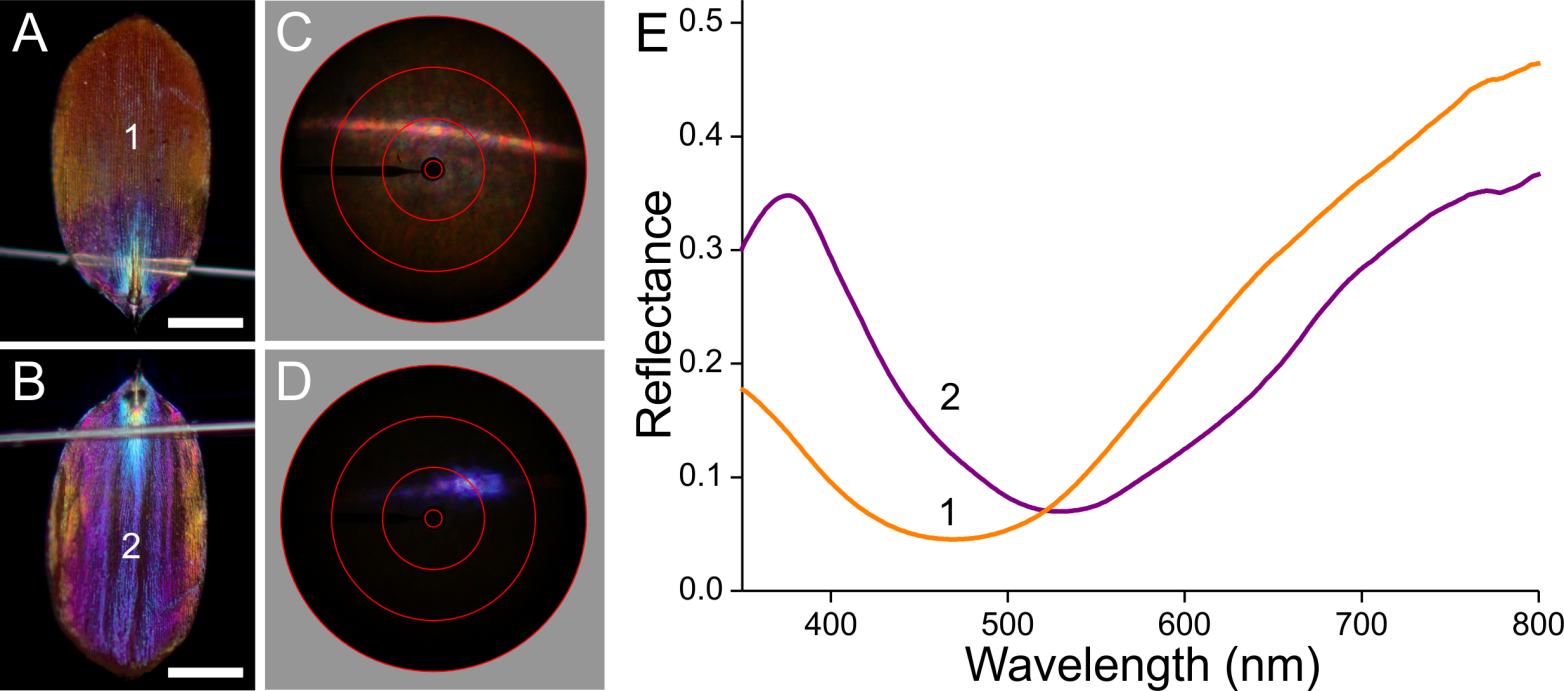
Scale of an orange hindwing area (Fig. 1, #4). (A, B) Photographs of the abwing and adwing sides (scale bars: 50 µm). (C, D) Scatterograms of the two sides. (E) Reflectance spectra of the numbered locations in (A) and (B).

To unravel the optical consequences of the scales’ pigmentation and structuring, we performed epi-illumination light microscopy (Figs. 6–9, A, B), imaging scatterometry (Figs. 6–9, C, D), as well as microspectrophotometry (Fig. 6E). Gluing a single, isolated scale to the tip of a glass micropipette and mounting the pipette to a rotatable manipulator allowed subtle adjustment of the scale. A scale of the white areas, which showed an almost pure white colour when the scale was still attached to the wing, becomes bluish when isolated from the wing. The central and tip areas of the abwing side (i.e., the side normally seen; Fig. 6A) are moderately bluish, but near the scale pedicel the blue colour intensifies. When inspected at the adwing side (facing the wing; Fig. 6B), a bright blue to violet colour emerges.

The scatterograms of the two sides of the ‘white’ scale strongly differ (Figs. 6C, 6D). The abwing scatterogram reveals a distinct line, due to diffraction at the array of ridges, together with a broad-angled scattering, due to the diffusive properties of the upper lamina. The scatterogram of the adwing side is a very localized blue spot, almost point-like, as expected from a reflecting mirror-like structure. Reflectance spectra measured from various locations of the scale (Fig. 6E) all show a clear maximum in the blue-violet at ∼420 nm and a minimum around 600 nm. Especially the spectra measured at the adwing side are strongly modulated, akin to thin-film reflectance spectra (Fig. 5). The abwing side spectra are much less modulated, in line with the abwing scatterogram, which showed that the upper lamina of the scale acts as a diffraction grating and a diffuser.

The black scale behaves optically similarly (Fig. 7). Due to the high melanin pigment content, the reflectance of the abwing side is very low (Fig. 7E, spectrum #1). With a substantial exposure time, the abwing scatterogram nevertheless features a clear ridge-based diffraction line (Fig. 7C). The adwing side on the other hand is brightly reflecting, and the scatterogram shows a very directional reflection, indicating again a mirror-like structure (Fig. 7D). The adwing colour, and thus the corresponding reflectance spectrum, strongly depends on the location, but the spectra all closely resemble thin-film reflectance spectra (Fig. 7E, #2–6).

The brown scale represents an intermediate case (Fig. 8). The abwing scatterogram shows a diffraction line as well as broad-angled scattering (Fig. 8C), while the point-like adwing scatterogram (Fig. 8D) demonstrates again a mirror-like reflection, well corresponding to the thin-film-like reflectance spectrum (Fig. 8E, #2). The abwing reflectance spectrum increases with increasing wavelength, characteristic for melanin pigmentation, but it has a noticeable bulge around 500 nm, precisely in the wavelength range where the adwing reflectance is high (Fig. 8E, #1). This can be readily interpreted as that the main component of the abwing reflectance is due to scattered light, spectrally filtered by the melanin of the upper lamina, and that a minor contribution is made by the blue–green reflecting thin film of the lower lamina.

The orange scale seems to be a rather aberrant case when compared to the previous cases (Fig. 9). When observed from the upper, abwing side (Fig. 9A), it appears that the main part of the scale is orange, but the root area near the pedicel is violet, alike the main adwing side (Fig. 9B). The abwing scatterogram shows the familiar diffraction line plus a broad-field scattering (Fig. 9C), and the adwing scatterogram is again locally restricted (Fig. 9D). The ab- and adwing reflectance spectra have similar shapes with deep valleys around 500 nm (Fig. 9E). The abwing scatterogram and reflectance spectrum confirm the presence of the strongly blue-absorbing ommochrome pigment in the upper lamina, and the adwing scatterogram and reflectance spectrum confirms the thin-film reflector of the lower lamina, which reflects strongly violet and red light, together causing the adwing’s purple colour.

### Reflectance spectra and thickness of the lower laminae and the wing substrate

The scatterograms demonstrate that the lower lamina of the wing scales acts as an optical thin film. Using the peaks and valleys of the reflectance spectra of the various investigated scales (Figs. 6–9), the local thickness of the lower lamina can be directly read from Fig. 5B. For instance, the adwing reflectance spectra indicate that the thickness of the lower lamina of the white scale (Fig. 6) is ∼210 nm; the thickness of the lower lamina of the black scale (Fig. 7) is near the pedicel ∼280 nm, gradually decreasing to the border, where the thickness is ∼180 nm; the thickness of the lower lamina of the brown scale (Fig. 8) is ∼240 nm; and that of the orange scale (Fig. 9) is ∼180 nm. The thickness values are approximate, because the thin films are clearly not perfect. For ideal thin films, the reflectance value in the minima is zero, which is not the case for the experimentally obtained spectra. Of course, the adwing reflectance spectra consist partly of a contribution by the upper lamina, but that is at least in the case of the black scales very minor. Although the spectral measurements were from micrometer-sized areas, we thus have to conclude that the demodulated reflectance spectra are due to local, sub-micrometre variations in the thickness of the lower lamina of the scales.

**Figure 10:**
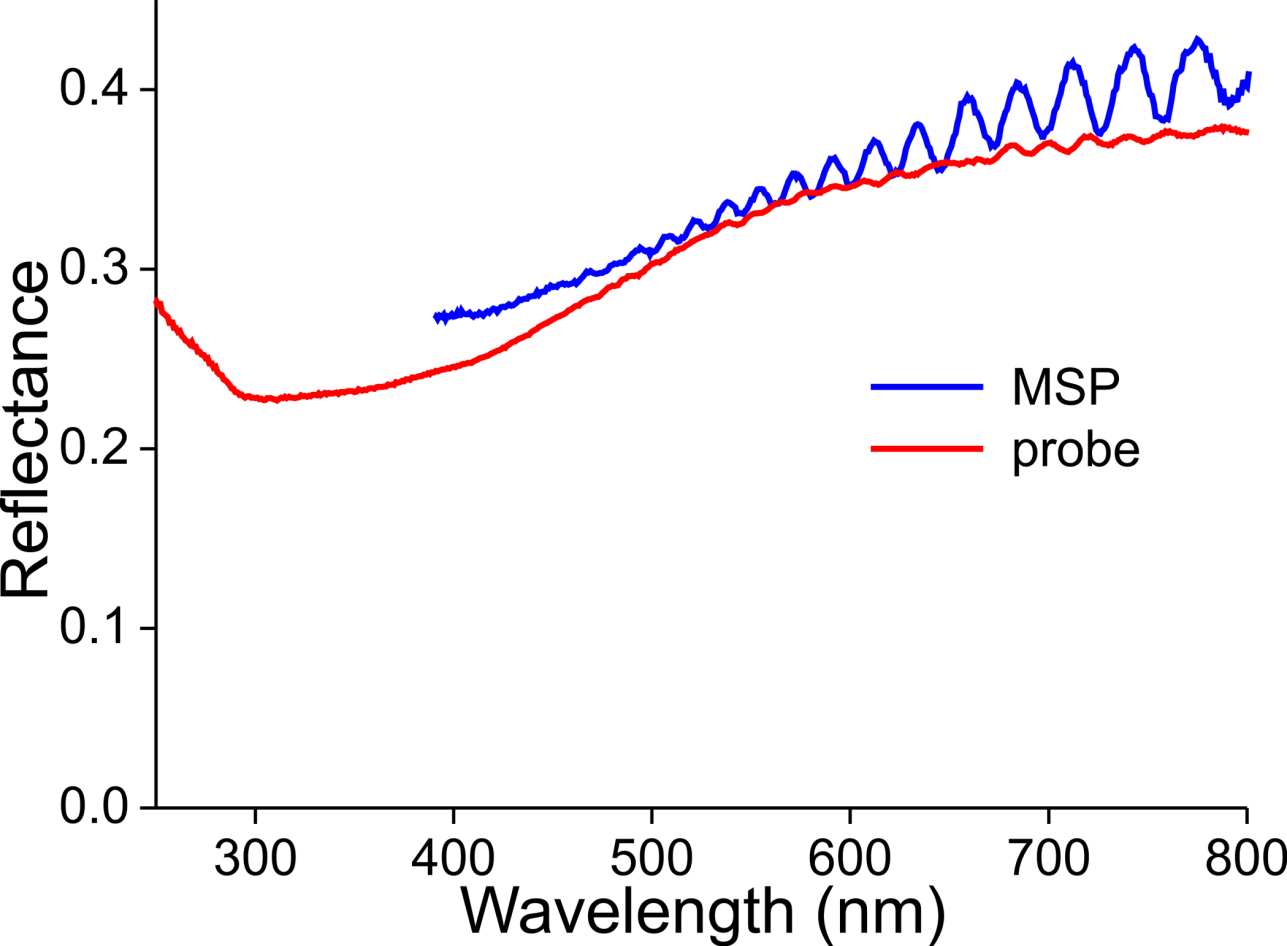
Reflectance spectra of a denuded wing membrane measured with a microspectrophotometer (MSP) and a bifurcated probe (probe).

The latter, a non-constant thickness, is very apparent when measuring the reflectance spectra of a wing membrane that was devoid of wing scales (Fig. 10). High frequency oscillations occur in the reflectance spectra obtained with both the microspectrophotometer and the bifurcated probe. As the measurement area of the probe is in the millimeter range, the amplitude of the oscillating reflectance (Fig. 9, red curve) is very small, because a varying membrane thickness then causes averaging. The thickness of the wing membrane can be derived from the oscillation frequency and the refractive index of chitin (Stavenga, 2014), yielding 5–8 µm.

### Scales on the wing

*In situ*, on the wing, the scales form a stack of each other overlapping scales. This allows light that has been transmitted by the scale on top of the stack to be reflected by a lower scale and/or by the wing membrane (and ultimately even by scales on the opposite side of the wing; Stavenga, Giraldo & Hoenders, 2006; Stavenga, Leertouwer & Wilts, 2014a). The reflectance spectrum of the stack of scales in the white areas hence becomes severely demodulated, although the distinct contribution of the blue-reflecting lower laminae nevertheless remains unmistakable (Fig. 1B).

**Figure 11:**
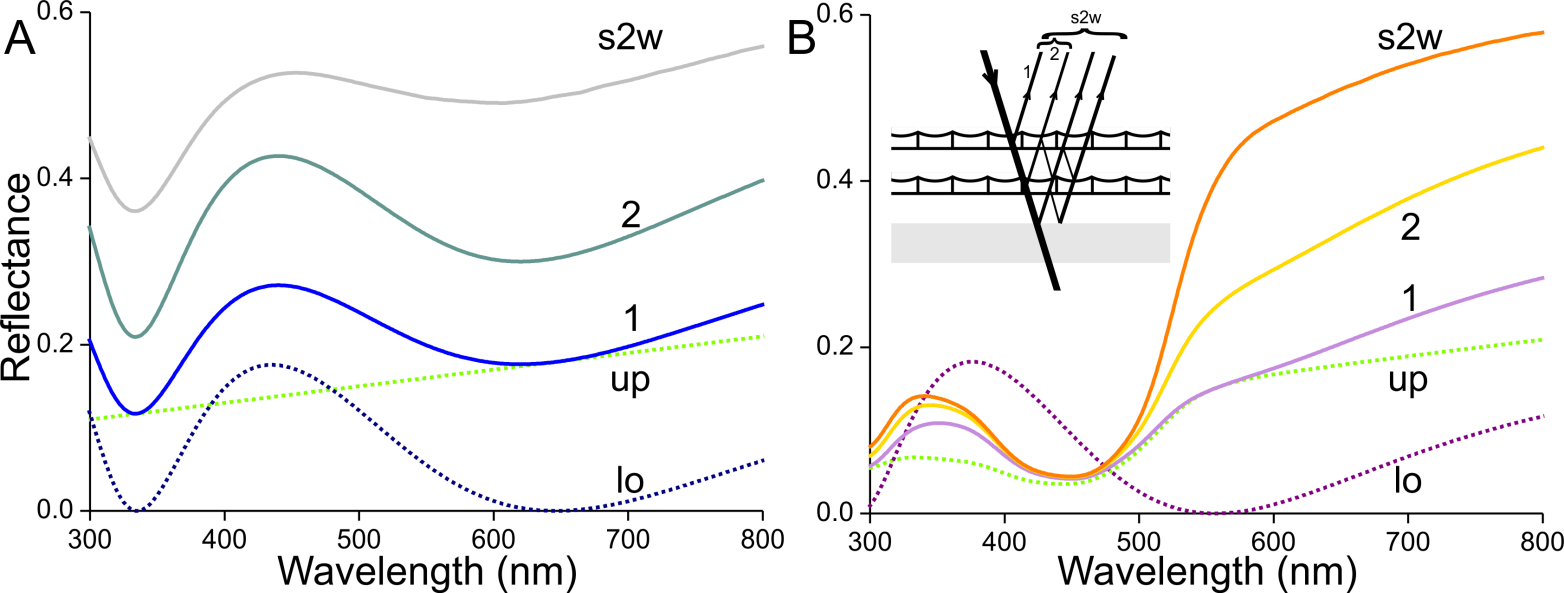
Modelling reflectance spectra of white and orange wing areas. (A) Reflectance spectra of one (1) unpigmented scale, of a pile of two (2) such scales, and of the latter stacked on the wing (s2w). (B) Reflectance spectra of one (1) scale containing the ommochrome pigment of Fig. 3C, of a pile of two (2) such scales, and of the latter stacked on the wing (s2w), see inset; up and lo: reflectance spectra assumed for the upper (up) and lower (lo) lamina of the single scales.

The optical consequences of scale stacking are visualized in Fig. 11 using a simple, heuristic model. First, we consider an unpigmented scale with an unstructured upper lamina and a thin-film lower lamina. Inspired by the measurements, and considering that the absorbance of transparent scales is not fully zero but decreases monotonically with increasing wavelength (Fig. 3B), we assume for the upper lamina a modest, linearly-rising reflectance spectrum, given by *R*_up_ = 0.14 + 2⋅10^−4^*λ* (*λ* in nm; Fig. 11A, up, dotted green curve). Using Eq. (1), a lower lamina with thickness 210 nm yields a blue-peaking reflectance spectrum, *R*_lo_ (Fig. 11A, lo, blue dotted curve; maximum 435 nm, minimum 645 nm, see also Fig. 5). Treating the single scale as a stack of two layers, i.e., the upper and lower laminae, its reflectance can be calculated with Eqs. (2) and (3), using *T*_up_ = 1 − *R*_up_ and *T*_lo_ = 1 − *R*_lo_ as the scale is unpigmented. The reflectance spectrum calculated for the single scale features a distinct blue peak (Fig. 11A, curve 1), which is however much less pronounced than the blue peak of the lower lamina. With a stack of two identical, unpigmented scales (thus with four layers) the resulting reflectance spectrum is again broad-band with a blue peak (Fig. 11A, curve 2), and adding the reflecting wing membrane (probe spectrum of Fig. 10) yields a distinctly further demodulated spectrum (Fig. 11A, curve s2w). The latter resembles the spectrum of the white wing area (Fig. 1, curve 1).

Next, we consider an orange scale. A lower lamina with thickness 180 nm, suggested by the adwing reflectance spectrum (Fig. 9E, curve 2), yields the reflectance spectrum of Fig. 11B (lo, purple dotted curve; reflectance maximum 375 nm, minimum 555 nm, see Fig. 5). Expressing in the scales an ommochrome pigment with the absorbance spectrum of Fig. 3C and an absorbance peak value of 0.6 (average of measured values) will suppress the reflectance (as well as transmittance) of the upper lamina in the blue wavelength range (Fig. 11B, up, dotted green curve). The reflectance spectrum calculated for the intact scale (the stack of upper and lower laminae) shows a slightly increased reflectance in the UV and red compared to the upper lamina’s reflectance spectrum (Fig. 11B, curve 1). A stack of two scales has a substantially increased reflectance, especially in the orange and red wavelength range (Fig. 11B, curve 2). Adding the reflecting wing membrane further enhances this wavelength-restricted reflectance increase (Fig. 11B, curve s2w). The latter reflectance spectrum again resembles the spectrum measured from the orange wing area (Fig. 1, curve 4).

The reflectance spectra of the brown and black scales, which are dominated by various amounts of melanin pigment, can be similarly modelled.

## Discussion

### Wing colouration and pigmentation

The giant butterfly-moth *Paysandisia archon* is a dayflying castniid moth with butterfly-like behaviour (Sarto i Monteys et al., 2012). Its dorsal hindwings, with the black-rimmed white spots in a colourful orange background, presumably function in the intraspecific recognition between males and females, mediated by a visual system that at the front end has extremely large compound eyes equipped with four classes of spectral photoreceptors, peaking at 360, 465, 550, 580 nm, respectively (Pirih et al., 2018, unpublished data).

The wings are populated by four main types of scales: white, black, brown, and orange. At least four optical mechanisms determine the scale colours: absorbing pigments (melanin and ommochromes), thin-film interference (lower lamina), ridge-array diffraction grating, and scattering (upper lamina). The wing colours are hence a mixture of pigmentary and structural colourations.

We found that the orange scales contain an ommochrome pigment. Its absorbance spectrum, peaking at ∼450 nm, virtually coincides with the absorbance spectrum of the ommochrome extracted from the orange wings of *Heliconius numata* (Wilts et al., 2017b). Ommochromes with very similar absorbance spectra were identified as ommatin-D and xanthommatin (Bolognese et al., 1988; Nijhout, 1997). The absorbance spectra of the two black scales in Fig. 3B substantially differ in the blue wavelength range, indicating the additional presence of the blue-absorbing ommochrome (Fig. 3C) in one of the scales. The absorbance spectrum of the brown scale in Fig. 3B shows a considerable increase in the short-wavelength range, possibly due to the UV-violet-absorbing kynurenine (e.g., Reed, McMillan & Nagy, 2008; Stavenga, Leertouwer & Wilts, 2014a).

### Wing scale optics

We have analysed the four types of scales that principally create the wing colours of *P. archon*. The anatomy, pigmentation as well as optical characteristics are similar to those of the scales of common, diurnally-active nymphaline butterflies (Stavenga, Leertouwer & Wilts, 2014a), but there are a number of critical differences. In the basic anatomy of nymphaline butterfly scales, the upper lamina mainly consists of an array of parallel ridges, with few crossribs leaving wide-open windows (Ghiradella, 2010). Light incident from the abwing side that hits the ridges is backscattered, which due to interference causes the linear diffraction pattern in scatterograms: the ridge array acts as a diffraction grating. Yet, with large windows, a substantial fraction of incident light reaches the lower lamina, and the light reflected by the lower lamina can thus in turn pass the upper laminar structures mainly unhampered. Consequently, the lower lamina can play an important role in determining the wing scale colour of nymphalines (Stavenga, Leertouwer & Wilts, 2014a).

Quite differently, in the upper lamina of the scales of *P. archon* the close packed crossribs (which are actually extensions of microribs; Ghiradella, 2010) leave negligible or at most very small windows, very similar as found in the scales of primitive lepidopterans as well as of more advanced species (Simonsen, 2001; Simonsen & Kristensen, 2001; Ingram & Parker, 2008; Stavenga, Leertouwer & Wilts, 2014b). The optical consequence is that light incident from the abwing side, in addition to being diffracted by the ridge array, will be reflected by the crossribs and the membrane in between them. Since that membrane is not flat, the latter reflected light will contribute a broad-band reflectance spectrum. In the case of the unpigmented scales, the light fraction transmitted by the upper lamina will contribute a blue-peaking reflectance of the thin-film lower lamina, causing a bluish-white scale colour (Fig. 6A).

In the black scales, the high concentration of melanin pigment fully determines the abwing reflectance spectrum, because the strongly absorbing melanin very effectively suppresses the reflected as well as transmitted light flux. Near the scale pedicel, the melanin concentration is small (Fig. 3A2), so that a substantial fraction of abwing incident light, after being transmitted by the upper lamina, is reflected by the lower lamina and then passes the upper lamina again, resulting in a coloured root area (Fig. 7A).

The optical behaviour of the brown scales can be readily understood also. The moderate amount of melanin in the upper lamina (Fig. 4A) acts as a weak optical filter, resulting in a rather indistinct colour. The blue–green reflecting lower lamina causes a slight upsurge of the reflectance spectrum around 500 nm (Fig. 8E).

The most interesting is of course the orange scale (Fig. 9). The extreme differences in the appearance as well as in the reflectance spectra of the abwing and adwing sides suggest that only the upper lamina elements contain ommochrome pigment. It there acts as a strong optical filter, which severely reduces the reflected as well as transmitted light by the upper lamina in the blue wavelength range. The pigmentation is poor in the root region (Fig. 3A4), causing the purple colour of that area at the abwing side (Fig. 9A). Interestingly, the reflectance spectrum of the lower lamina seems to almost match the upper lamina’s spectrum, as it also reflects little in the blue and reflects strongly in the ultraviolet as well as longer wavelength range (Fig. 9E). The long-wavelength (and ultraviolet) light that is transmitted by the upper lamina can thus be substantially reflected by the lower lamina and hence contribute to the scale colour.

### Scales stacked on the wing

We heuristically modelled the reflectance spectra of stacks of scales *in situ* (Fig. 11). In the white wing areas, a pile of unpigmented, blue scales yields a broad-band reflectance spectrum; an increasing number of stacked scales on the wing causes a desaturated colour contrast. In the orange wing areas, a stack of scales filled with ommochrome pigment creates a distinct orange colour. The reflectance of the stacked orange scales remains low in the blue wavelength range, but in the long-wavelength range the cumulative reflectance of the overlapping scales as well as the wing substrate causes a sharply increasing reflectance around 600 nm (Fig. 1B). Hence, in this case an increasing number of superimposed scales causes an increased colour contrast. We may note that the orange scales have a lowly pigmented root area, but that part is *in situ* overlapped by another scale (Fig. 2C). The lower lamina then will substantially contribute to the reflectance, so there is no serious drawback for the reflectance in the long-wavelength range.

The same holds for the black scales. They also have less pigment in the root area (Fig. 3A2), resulting in a blue shine when inspecting the abwing side of a single scale (Fig. 7A). The main area is heavily pigmented, and as the black scales *in situ* also overlap each other, the reflecting root area is well covered. The low reflectance of the single scales and their considerable overlap on the wing causes the extremely low reflectance of the black wing areas compared to the white and orange wing areas, thus creating a starkly contrasting wing pattern (Fig. 1).

Overlap of the brown scales on the forewing is rather limited (Fig. 2B). As a result, light transmitted by the scales is partly reflected by the wing membrane and then again in part transmitted by the scales, increasing specifically the long-wavelength reflectance of the brown areas of the forewing. The reflectance spectrum of the assembly of brown scales on the wing thus obtains a steeper slope in the long-wavelength range (Fig. 1B).

Scale stacking is a common characteristic of lepidopteran wings, and the optical effects discussed above are indeed generally applicable. For instance, identically to described above, in the white wing areas of nymphalines the scales are unpigmented and blue reflecting, but stacked upon each other and the wing substrate they cause a broad-band reflectance, also with a hump in the blue wavelength range. However, in the blue eyespots in the hindwings of the peacock *Aglais io*, the blue scales are stacked upon black scales, which preserves the upper scale’s blue colour (Stavenga, Leertouwer & Wilts, 2014a; Siddique et al., 2016). This organization has been realized in many butterfly species, but not in *P. archon*.

## Conclusions

The wings of the giant butterfly-moth *Paysandisia archon* are covered by four main types of scales. The scale colours, which have both a pigmentary and structural basis, can be described by a few optical principles that universally apply to the scale lattice covering lepidopteran wings. The scales’ upper lamina acts as a diffuser and a diffraction grating, the lower lamina is a thin-film interference reflector. Pigments expressed in the scales act as spectral filters.

##  Supplemental Information

10.7717/peerj.4590/supp-1 Data S1 Dataset

## Additional Information and Declarations

### Data Availability

The following information was supplied regarding data availability: All data needed to evaluate the conclusions in the paper are present in the paper, and raw data are provided in a Supplemental File. Additional data related to this paper may be requested from the authors.
